# HDAC3 Inhibitor RGFP966 Modulates Neuronal Memory for Vocal Communication Signals in a Songbird Model

**DOI:** 10.3389/fnsys.2017.00065

**Published:** 2017-09-05

**Authors:** Mimi L. Phan, Mark M. Gergues, Shafali Mahidadia, Jorge Jimenez-Castillo, David S. Vicario, Kasia M. Bieszczad

**Affiliations:** Department of Psychology, Behavioral & Systems Neuroscience, Rutgers, The State University of New Jersey New Brunswick, NJ, United States

**Keywords:** epigenetics, memory, NCM, stimulus specific adaptation (SSA), electrophysiology, gene expression, lateralization

## Abstract

Epigenetic mechanisms that modify chromatin conformation have recently been under investigation for their contributions to learning and the formation of memory. For example, the role of enzymes involved in histone acetylation are studied in the formation of long-lasting memories because memory consolidation requires gene expression events that are facilitated by an open state of chromatin. We recently proposed that epigenetic events may control the entry of specific sensory features into long-term memory by enabling transcription-mediated neuronal plasticity in sensory brain areas. Histone deacetylases, like HDAC3, may thereby regulate the specific sensory information that is captured for entry into long-term memory stores (Phan and Bieszczad, 2016). To test this hypothesis, we used an HDAC3-selective inhibitor (RGFP966) to determine whether its application after an experience with a sound stimulus with unique acoustic features could contribute to the formation of a memory that would assist in mediating its later recognition. We gave adult male zebra finches limited exposure to unique conspecific songs (20 repetitions each, well below the normal threshold to form long-term memory), followed by treatment with RGFP966 or vehicle. In different groups, we either made multi-electrode recordings in the higher auditory area NCM (caudal medial nidopallidum), or determined expression of an immediate early gene, *zenk* (also identified as *zif268*, *egr-1*, *ngfi-a* and *krox24*), known to participate in neuronal memory in this system. We found that birds treated with RGFP966 showed neuronal memory after only limited exposure, while birds treated with vehicle did not. Strikingly, evidence of neuronal memory in NCM induced by HDAC3-inhibition was lateralized to the left-hemisphere, consistent with our finding that RGFP966-treatment also elevated *zenk* expression only in the left hemisphere. The present findings show feasibility for epigenetic mechanisms to control neural plasticity underlying the formation of specific memories for conspecific communication sounds. This is the first evidence in zebra finches that epigenetic mechanisms may contribute to gene expression events for memory of acoustically-rich sensory cues.

## Introduction

The brain has a remarkable ability to encode, represent and remember the sensory details of experiences. Stimuli with adaptive significance can be remembered for periods of time that can last even as long as a lifetime. Moreover, such long-term memories can possess a high degree of stimulus-specificity that allows memory to be discriminative between similar, but non-identical, stimuli and events, despite only subtle differences between them. How does the brain encode and store such highly detailed sensory information? Songbirds provide a powerful model for investigating memory mechanisms because their brains are specialized to encode and remember the songs of other individuals, which are unique in the details of their acoustic features. In the zebra finch caudal medial nidopallidum (NCM), neurons show selective responses and preferential, long-lasting neuronal memory for conspecific vocalizations (Mello et al., 1992; Chew et al., 1996a,b; Bolhuis and Gahr, 2006; Phan et al., 2006; Bell et al., 2015; Elie and Theunissen, 2015). A critical factor that contributes to the formation of a neuronal memory in NCM for a unique song is the amount of exposure to that particular song, which can be experimentally parameterized by the number of repetitions (Mello et al., 1995; Chew et al., 1996a,b; Stripling et al., 1997). The bird must hear a minimum number of repetitions of a unique conspecific birdsong to form a lasting memory of that song. Furthermore, the neural mechanisms that control the induction of neural plasticity with increasing exposure depend on precisely timed and coordinated waves of gene expression across repetition number (Mello et al., 1992; Mello and Clayton, 1994; Chew et al., 1995, 1996a,b; Stripling et al., 2001; Hahnloser and Kotowicz, 2010). Thus, mechanisms that control transcription may be key for setting the threshold exposure required to release the brakes on gene expression that are required to induce plasticity in NCM and provide the substrate for long-term memory of a unique song.

*De novo* gene expression is known to underlie long-lasting neural plasticity that can store learned information over time in many systems, including invertebrates, mammals and birds (Alberini, 2009). It is now becoming more widely accepted that experience-dependent instances of transcriptional activation may be gated by epigenetic mechanisms (Roth et al., 2010; Day and Sweatt, 2011). Electrophysiological and behavioral correlates of memory have been shown to rely on neuroplasticity mediated by epigenetic mechanisms (Rudenko and Tsai, 2014; Spiegel et al., 2014; Cholewa-Waclaw et al., 2016). Moreover, work in rodents (McQuown et al., 2011; Day et al., 2013; Malvaez et al., 2013; Bieszczad et al., 2015), in bees (Biergans et al., 2012, 2015, 2016), and invertebrates (Federman et al., 2009, 2014) shows that two epigenetic mechanisms in particular, histone acetylation and DNA methylation, can alter the stimulus-specificity of sensory information that is captured by memory consolidation processes. Stimulus-specific consolidation may enable feature detection and discrimination of the remembered sensory stimulus over time scales that indicate long-term memory storage, e.g., 24 h after the stimulus experience (e.g., Korzus et al., 2004; Vogel-Ciernia and Wood, 2014).

Epigenetic marks like methyl-groups added to the DNA backbone or acetyl-groups covalently bound to lysine tails of histones in the context of chromatin, can appear near specific subsets of genes to gate their transcription. The histone deacetylases (e.g., HDAC3) for example, oppose the action of histone acetylases (e.g., like CREB-binding protein, CBP), to act as a molecular brake on neuronal memory formation by removing acetyl groups thereby constricting access to chromatin at select gene promoters resulting in silencing of transcription (Ruthenburg et al., 2007; Day and Sweatt, 2011). Therefore, a HDAC3-inhibtor can act to “release the molecular brakes” on gene expression events required for consolidation (McQuown and Wood, 2011; Vogel-Ciernia and Wood, 2012; Malvaez et al., 2013; White and Wood, 2014). Pharmacological inhibitors of HDACs have been useful tools to determine how histone acetylation plays a role in neuronal and behavioral memory (Stefanko et al., 2009; McQuown et al., 2011; Malvaez et al., 2013; Bieszczad et al., 2015).

By using the HDAC3-selective inhibitor, RGFP966, we aimed to determine whether memory for complex stimuli with unique acoustic features can be controlled by a molecular epigenetic mechanism. Notably, NCM neurons categorically respond to distinct, complex conspecific vocalizations, do so selectively, and have potential for experience-dependent plasticity. Hereafter, we call NCM plasticity—in the form of long-lasting song-selective neurophysiological adaptation—“neuronal memory” and use this as neurophysiological evidence to determine RGFP966 effects on the formation of memory for unique conspecific birdsongs. If RGFP966 can lower the song-exposure threshold for the formation of song-specific neuronal memory in NCM, then HDAC3 may function as a molecular brake on discriminative memory formation for complex stimuli with distinctive acoustic features.

## Materials and Methods

### Animal Subjects

All procedures were approved by the AAALAC accredited Institutional Animal Care and Use Committee (IACUC) at Rutgers University as per guidelines in use according to Public Health Service Policy, recommendations and guidelines contained in the Guide for the Care and Use of Laboratory Animals, and applicable provisions of the Animal Welfare Act. Adult male zebra finches (*Taeniopygia guttata; N* = 32 either bred in our aviary or purchased from a commercial vendor were used in this study. Electrophysiological assessment of neuronal memory was determined using a comparison between birds with limited auditory stimuli presentations (i.e., a single conspecific song repeated 20 times within the stimulus set, “20X”), who were randomly assigned to either the group treated with an HDAC3-inhibitor, RGFP966 (“HDAC3-i”; *N* = 8) or to the group treated with vehicle (“Vehicle”; *N* = 6). These groups were compared to a group presented with an extended exposure to conspecific songs (i.e., a single conspecific song repeated 200 times within the stimulus set, “200X”, *N* = 6).

Additional adult male birds were used for immunohistochemical (*N* = 2) or gene expression analyses (*N* = 10). Immunohistochemistry of HDAC3 was performed on one bird sample from each training condition studied to confirm the nuclear presence of a Class 1 HDAC like HDAC3. For the gene expression studies, brain samples were from birds that had been randomly assigned to treatment with drug or vehicle with 20X limited training.

### Electrophysiological Recordings from NCM and Non-NCM Brain Regions

Methods for neurophysiological recording are as described in Phan et al. (2006); Phan and Vicario (2010); Tsoi et al. (2014) and Bell et al. (2015). Briefly, the subject underwent surgical preparation for the neural recording prior to the presentation of auditory stimuli used for electrophysiological assessment of evoked responses to sound. Each bird was anesthetized (1.5%–2.0% isoflurane in oxygen), and surgically implanted with a head fixation pin and recording chamber over a craniotomy centered over the auditory forebrain using dental cement (Dentsply Caulk, Milford, DE, USA). A motorized microdrive (Eckhorn, Thomas Recording, Giessen, Germany) was used to advance independently 16 tungsten microelectrodes (quartz platinum/tungsten, impedance: 2–4 MΩ, Thomas Recording) bilaterally into the brain, targeting areas NCM (four electrodes per hemisphere) and the Field L complex (four electrodes per hemisphere; consisting of L1, L2a and L3; referred to as Non-NCM in the text). Both areas were defined prior to electrode placement from fiduciary landmarks centered on the bifurcation of the midsagittal sinus. Additionally, NCM and non-NCM areas were located electrophysiologically by their characteristic response patterns to white noise search stimuli shaped with the amplitude envelope of zebra finch song.

### Song Exposure and Test of Neuronal Memory for Song

To measure the effect of song exposure (20X or 200X) on neuronal memory for songs, the awake bird was exposed to 20 (20X) or 200 (200X) blocked playbacks of eight conspecific songs. The exposure paradigm repeated each of the eight songs either 20 times (20X, blocked, ISI 8 s; “limited” exposure condition) or each of the eight songs 200 times (200X, blocked, ISI 8 s; “extended” exposure condition). Twenty hours later, electrophysiological recordings from NCM and non-NCM brain regions were used to determine the effect of prior exposure on neuronal memory.

Neuronal memory was assessed in a large walk-in acoustically isolated sound booth (IAC, Bronx, NY, USA) in the awake bird 20 h after song exposure. The bird was gently restrained in a custom body tube, with the head pin clamped to a stereotaxic apparatus for stable recordings. Extracellular multi-unit activity was simultaneously recorded from all 16 electrodes located in NCM or non-NCM, amplified (total gain: 19,000), bandpass filtered (0.5–5 kHz) and digitized (25 kHz per channel) for further analysis, as previously described (Phan and Vicario, 2010; Bell et al., 2015). Specialized software (Spike 2, Version 7, CED, Cambridge, UK) was used to deliver song stimuli and record neural activity. Four sets of song stimuli were used to test for neuronal memory, each consisting of two conspecific songs from the exposure set, called “familiar” (F) and two completely novel conspecific songs (“novel”, N). Each set included 25 repetitions of each song in shuffled order (8 s interstimulus interval; sets were presented immediately one after another in the same session). The bird thus heard a total of 8F and 8N song presentations in the recording session.

### Histone Deacetylase Inhibitor and Administration Protocol

Birds were injected immediately following the 20X song exposure session. The bird was restrained by hand and given a systemic injection of either RGFP966 (10 mg/kg, i.m.) or vehicle (i.m.) into the pectoralis muscle, and returned to their home cage, located inside the sound booth. The effective systemic 10 mg/kg dose for memory modulation has been established in rodents (Malvaez et al., 2013; Bieszczad et al., 2015). Furthermore, RGFP966 (10 mg/kg) can penetrate the blood-brain-barrier within 15 min and its concentration in the brain begins to decline by 120 min, as established in rodents (Malvaez et al., 2013; Bieszczad et al., 2015). Following RGFP966, hereafter called “HDAC3-i”, or vehicle administration, the bird remained in its cage inside the recording booth until the electrophysiological assessments for neuronal memory 20 h later.

### Electrophysiological Analysis for Detecting Neuronal Memory

**Figure 1:**
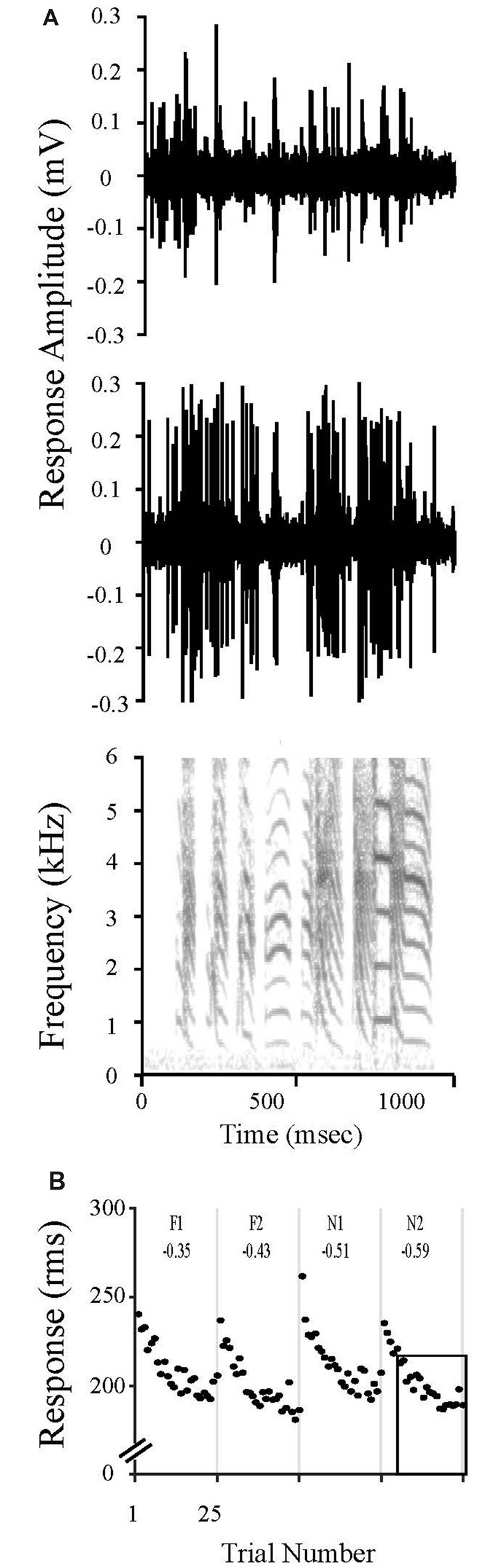
Stimulus Specific Adaptation (SSA) to song stimuli is evident in caudal medial nidopallidum (NCM) electrophysiological responses. **(A)** Multi-unit recordings from two unique sites in NCM show evoked responses to a zebra finch song. The sound spectrogram is representative of the stimuli presented during to evoke electrophysiological responses. Note the complex features in the sound stimulus, such as the differences in fundamental frequencies and harmonic acoustic structures that vary among the individual syllables. These features distinguish each song motif, resulting in unique identifiers for an individual bird vocalization. **(B)** NCM neuronal responses change with repeated exposures to the same song stimulus. The response magnitude of multiunit activity recorded at one site in NCM are plotted over time during the presentation of songs that were either previously heard during the limited song exposure (20X; F1, F2) or that were novel (N1, N2). Both stimulus classes were presented 20 h after the limited song exposure. Each unique song was played 25 times in shuffled order, but responses have been reordered in the plot for clarity. SSA rates were always calculated for trials 6–25 as indicated by the box outline in N2 (see “Materials and Methods” Section “Electrophysiological Analysis for Detecting Neuronal Memory”) and are shown at the top of each panel.

Neurons in NCM undergo a dynamic process of stimulus specific adaptation (SSA) in which repeated presentations of an initially novel stimulus evoke progressively smaller responses to an asymptote; however, robust responses return upon presentation of a new song stimulus (Chew et al., 1995, 1996a,b; Stripling et al., 1997; Phan et al., 2006). These reductions in responses occur specifically and independently for each stimulus. SSA lasts hours to days for conspecific vocalizations (Chew et al., 1996a,b; Phan et al., 2006; Bell et al., 2015; Yang and Vicario, 2015; Yoder et al., 2015). In contrast, SSA lasts only ~3 h for human speech and canary songs; 6.5 h for Bengalese songs. Pure tones presented in isolation do not evoke this class of SSA (Terleph et al., 2006). The SSA rate, which quantifies this property, is the slope of the decrease in response amplitude as a function of stimulus repetition number. Thus, stimuli that are novel, never before heard, show a high rate of adaptation across stimulus presentations, whereas familiar, remembered stimuli show a low rate of adaptation since NCM is already adapted to that song. Using established regression methods, we calculated the SSA rate as the slope of the decrease in response magnitude with successive presentations over the linear region of the adaptation profile (trials 6–25) at each electrode for each stimulus (Chew et al., 1995; Phan et al., 2006). The rate measure is normalized by the absolute amplitude to correct for response amplitude differences between recording sites (Chew et al., 1995; Phan et al., 2006). The resulting rates are negative slopes that quantify stimulus familiarity (the steeper the rate the less familiar or remembered; the shallower the rate, the more familiar; Figure 1).

The memory for songs heard 20 h prior was measured with established methods that use the SSA rates of NCM multiunit neuronal responses to familiar and novel song playbacks (Phan et al., 2006; Yoder et al., 2012, 2015; Tsoi et al., 2014). The 20 h designation for a “long-term memory” time-point is based on time-course studies that revealed the relationship between conspecific song exposure and the persistence of long term adaptation over time. The 20 h time-point is a time that requires a prior molecular consolidation event (i.e., both new RNA and protein synthesis) for neuronal memory to occur (Chew et al., 1996a,b). Furthermore, the “extended” 200X exposure condition is known to result in robust SSA measured at the 20 h time mark (Chew et al., 1996a,b; Tsoi et al., 2014). This also adheres to standard behavioral definitions of long-term memory, which is typically tested ~24 h after a learning experience (Korzus et al., 2004; Vogel-Ciernia and Wood, 2014). A “Familiarity Index” (FI) is calculated by dividing the adaptation rate to novel songs by the rate to a given familiar song (N/F). When FI > 1, adaptation to a familiar song is slower than the adaptation rate to novel song, indicating a recognition memory. When FI = 1, the adaptation rate to the familiar song is equal to that of novel song, indicating that the song is processed as if novel, thus there is no detectable song memory (Phan et al., 2006; Yoder et al., 2015). FI values were determined for each recording site. All sites within the same hemisphere, brain region and/or training condition were pooled to produce a median FI value. The sample sizes for each pooled dataset were as follows; NCM: FI *N*_drug_ = 162; FI *N*_vehicle_ = 124; non NCM areas: FI *N*_drug_ = 179; NCM: Left-hemisphere FI *N*_drug_ = 97; Left FI *N*_vehicle_ = 63; NCM: Right-hemisphere FI *N*_drug_ = 65; Right FI *N*_vehicle_ = 61.

### Immunohistochemistry Studies

Two birds were euthanized with isoflurane, quickly decapitated, the skull was cut in half along a mid-sagittal section and the brain was rapidly excised from the skull. Each hemisphere was individually placed into a tissue embedding mold, covered with TissueTek (VWR, Radnor, PA, USA) and flash frozen in a slurry of 2% isopropanol and dry ice. The embedded brain was stored at −80°C until sectioned parasagittally on a cryostat in 10 μm thickness in further processing (Saldanha et al., 1999; Velho et al., 2007). The sections were thaw-mounted onto Superfrost slides (Fisher Scientific, Pittsburgh, PA, USA). Slides were fixed in cold 4% PFA, washed in 1× PBS, and blocked for 1 h in 8% NGS with 0.1% Triton X-100 in 1× PBS. Immunoreactions were with primary incubations in rabbit anti-NeuN (1:1000, EMD Millipore) and mouse anti-HDAC3 (1:1250, Cell Signaling Technologies). This HDAC3 antibody is reactive across human, monkey and rodent (Catalog#-3949, Cell Signaling Technologies). This is a monoclonal antibody produced using recombinant human HDAC3 that is expected to interact across mammal and avian species. A BLAST sequence analysis identified nearly complete overlap of the HDAC3 gene between birds and mammals, supporting the selectivity for an HDAC3-targeted antibody across both species. The zebra finch query sequence for HDAC3 protein (XP_012431694.1) was predicted from amino acid sequence to be 97% identical to *Rattus norvegicus* (AAH_61988.1); as well as 97% identical to *Homo sapiens* (NP_003874.2). Slides were incubated at 4°C on an orbital shaker overnight. Slides were subsequently washed and incubated for 2 h at room temperature with goat anti-mouse Cy3 (1:200, Sigma Aldrich), goat anti-rabbit FITC (1:1000, Sigma Aldrich) and counter-stained with DAPI (1:15000, Invitrogen). Each slide was imaged an EVOS-FL Auto 2.0 microscope (Thermofisher, Pittsburgh, PA, USA) in order to visualize localization of HDAC3 in the NCM region. Images were taken from the caudal dorsal area of the NCM^1^. Images were captured at either 40× magnification to obtain a stitched whole brain image; or at 100× magnification using an oil-immersion objective lens.

### Gene Expression Studies

Immediately following 20X song exposure, birds received a systemic injection of either HDAC3-i (RGFP966; *N* = 5) or Vehicle (*N* = 5). Individual left and right hemispheres were collected from each bird after 30 min. Individual NCMs were dissected out of the brain and for a subset of birds (RGFP966: *N* = 3; Vehicle *N* = 3) the Anterior Pole (AP) was also dissected out and quickly flash frozen in 2% isopropanol on dry ice. The rationale to use the AP was to compare gene expression with a region not involved in auditory processing and thus not likely to be affected by the song experience training protocol. Samples were dounce homogenized and total RNA was extracted with PureLink RNA Mini Kit (Invitrogen, Pittsburgh, PA, USA) as per manufacture’s protocol; 400 ng of RNA was then converted to cDNA using Transcriptor First Strand cDNA Synthesis Kit (Roche Diagnostics Co., Indianapolis, IN, USA) with thermocycler conditions: 10 min at 25°C, 30 min at 55°C, then 5 min at 85°C. For quantitative analysis, 1 μl of cDNA product was used for the qRT-PCR reaction using Power SYBR (Thermofisher, Pittsburgh, PA, USA) with primers for *c-fos* (forward—AGCTGGAGGAGGAGA AGTCC, reverse—CTCCTCGGAGAAGCACAACT), *zenk* (forward—ACTTCATCATCGCCATCCTC, reverse-TGGAATT GGGAAATGTTGGT), and *18 s (forward—CGAAAGCATTTG* CCAAGAAT, reverse—GGCATCGTTTATGGTCGG; Olias et al., 2014). Samples were run on a QuantStudio3 thermocycler (Thermofisher, Pittsburgh, PA, USA) with cycling parameters as follows: holding stage, 50°C for 2 min, 95°C for 10 min, and cycling stage: 95°C for 15 s, then 60°C for 1 min for 36 cycles. Relative gene expression levels were calculated using the ∆∆CT method by normalizing to housekeeping gene (i.e., *18s*) and was presented as percent of expression relative to vehicle controls.

### Statistics

Nonparametric Kolmogorov-Smirnov tests were used to determine significant differences in FI distributions between HDAC3-i and vehicle treated animals (OriginPro, OriginLab, MA, USA). Signtest for determining significant differences in FI from “1.0” to indicate the formation of memory (Matlab, Mathworks, MA, USA; Statistics and Machine Learning Toolbox; (Z) statistic available for sample sizes greater than 100). Nonparametric analyses were used to account for non-normal distributions of FI data. *T*-tests or ANOVAs were used in cases of normally distributed data. As for the gene expression studies, one-sample *t*-tests were used and Bonferroni corrected for multiple *t*-tests. For all tests, the alpha value was *α* = 0.05.

## Results

### Minimal Song Exposure Can Induce Long-Term Memory Formation in NCM with HDAC3-Inhibition

The main goal of these studies was to determine whether the experience threshold for long-term memory formation (tested 20 h later) could be reduced by administration of RGFP966, a selective inhibitor for HDAC3. We tested this by exposing birds to 20 repetitions of novel songs followed by either HDAC3-i or vehicle systemic injections, and then assessing song memory electrophysiologically 20 h later, following time-points of an established paradigm (Tsoi et al., 2014). Song-selectivity was measured electrophysiologically in NCM using a “familiarity” (FI) index, which, when greater than 1.0, indicates selective neuronal memory for *familiar, remembered* songs relative to *novel*, never-before-heard songs (*FI* = N/F). Birds treated with the HDAC3-i immediately after 20X exposure (*N* = 8) had significantly greater FI indices than birds with the same 20X exposure treated with vehicle (*N* = 6), indicating the formation of stronger song-selective memory 20 h after exposure (median *F*I_hdac3-i_ 1.19 > *F*I_veh_ 1.08; K-S *d*_stat_ = 0.163, *p* = 0.04). Furthermore, in vehicle injected birds, the FI showed no significant evidence of recognition memory 20 h later, (*F*I_veh_, *Z*_signtest_ = 2.25, *p* = 0.05; Bonferroni corrected), as predicted for this limited 20X exposure, based on earlier work (Chew et al., 1996a,b). In contrast, HDAC3-i injected birds had an FI significantly greater than 1.0 (*F*I_hdac3-i_, *Z*_signtest_ = 3.85, *p* = 0.00024).

**Figure 2:**
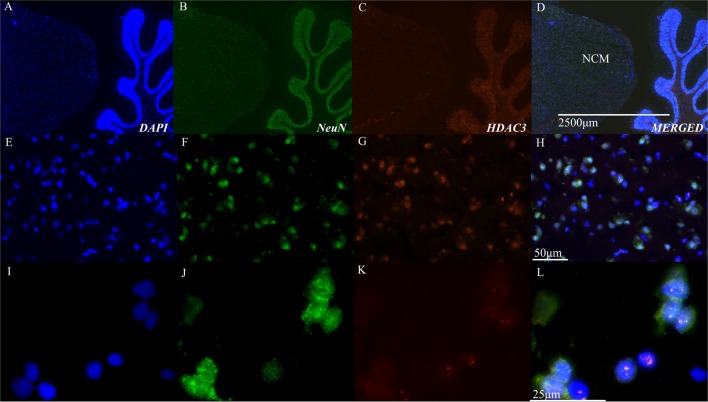
Histone deacetylase 3 (HDAC3) is evident in nuclei of NCM cells. **(A–D)** Representative sagittal section of NCM taken 0.75 mm from the midline.* Top row*: 40× magnification, scale bar = 2500 μm; *Middle row*: 40X magnification, scale bar = 50 μm; *Bottom row*: 100X magnification, scale bar = 25 μm. Columns show immunolabeling with antibodies against DAPI, NeuN, or HDAC3 as indicated. Last column shows a merged image. Images are from the target area of NCM at higher resolution **(E–H)** and magnification **(I–L)**. NCM, caudal medial nidopallidum.

These physiological results suggested a role for HDAC3 activity in the songbird brain. Immunohistochemical studies using neural markers and antibodies to HDAC3 support the physiological findings, which demonstrate that HDAC3 is indeed present in the bird brain, and appears inside NCM cells, including neurons (Figure 2).

### HDAC3-Inhibition Enables Neuronal Memory Selectively in NCM

To determine whether the HDAC3-i effect on neuronal memory was selective to the auditory forebrain region NCM, we compared FI values obtained from two auditory responsive regions in the avian forebrain: the NCM region, which is known to show FI > 1.0 with extended exposure (i.e., 200×) and the non-NCM region (Field L complex—L1, L2a and L3), which typically does not show FI changes with song exposure. In birds treated with HDAC3-i, FI values are significantly greater in NCM than non-NCM responses to song (median NCM *F*I_hdac3-i_ = 1.189; non-NCM *F*I_veh_ = 0.983; K-S *d*_stat_ = 0.232, *p* = 0.00016), with the FI significantly greater than 1.0 only in NCM (NCM *F*I_hdac3-i_, *Z*_signtest_ = 3.85, *p* = 0.00024; non-NCM *F*I_hdac3-i_, *Z*_signtest_ = 0.825, *p* = 0.819; Bonferroni corrected). Therefore, HDAC3-i did not affect auditory brain regions that normally do not participate in song-selective long-term memory formation. Rather, HDAC3-i effects appear to be selective for a brain area that normally participates in storing learned information about conspecific communication signals.

### Effects of HDAC3-Inhibition on Memory Formation in NCM are Lateralized

**Figure 3:**
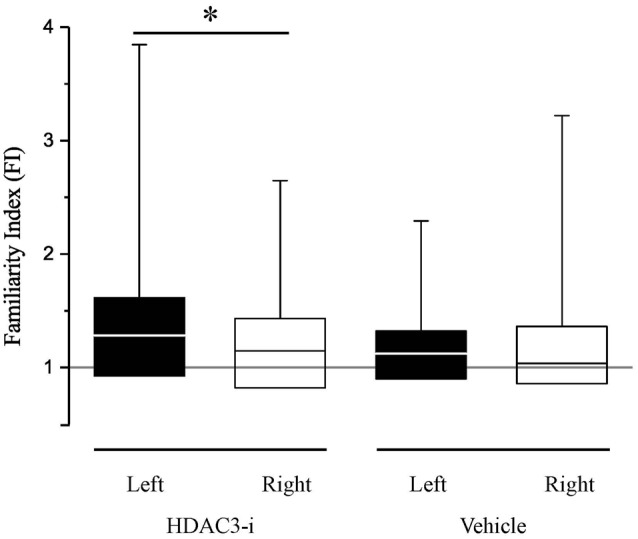
HDAC3-i enabled long-term neural memory in NCM, measured after only limited song exposure (20X), is lateralized. The Familiarity Index (FI) is a measurement of specific song memory derived from the neuronal responses in the NCM. An FI ratio at 1 (gray line) indicates that the previously exposed songs are not distinguished from novel, never before heard songs; an FI ratio > 1 indicates that the previously exposed song is more familiar (remembered) than the novel songs. FI measurements are significantly lateralized with a bias to higher FI’s in the left hemisphere in HDAC3-i treated birds (Kolmogorov-Smirnov Test, *d*_stat_ = 0.223; *p* = 0.03; median_left_ = 1.28; *IQR*_left_ = 0.69; *median*_right_ = 1.15; *IQR*_right_ = 0.61). In contrast, vehicle treated birds did not show a significant lateralization (Kolmogorov-Smirnov Test, *d*_stat_ = 0.162, *p* = 0.341). In the box and whisker plot, the box extends from the 25th to 75th percentiles, the line in the middle indicates median, and upper whiskers report maximum data values. *Indicates significant difference between groups, alpha = 0.05.

Bilateral recordings allowed the comparison of HDAC3-i effects between hemispheres, assessing memory with the FI as described above. When the FI indices between left- and right-NCM were compared, there was a significant difference (Figure 3), with a higher FI in the left hemisphere (median left-NCM *F*I_hdac3-i_ = 1.28; right-NCM *F*I_hdac3-i_ = 1.15; K-S *d*_stat_ = 0.223, *p* = 0.03). This is a strong lateralization effect, with memory measured only in the left hemisphere with a FI index significantly greater than 1.0 (left-NCM *F*I_hdac3-i_, *p* = 0.00098; right-NCM *F*I_hdac3-i_, *p* = 0.163; Bonferroni corrected). This indicates that the observed effect of HDAC3-i is driven by neuronal memory formation in the left hemisphere. This intriguing result adds to our knowledge of lateralization in songbird NCM, where auditory processing is known to show hemispheric differences (Phan and Vicario, 2010; Remage-Healey et al., 2010; Moorman et al., 2012; Tsoi et al., 2014; Chirathivat et al., 2015; Yang and Vicario, 2015).

To determine whether the reported lack of memory formation in the vehicle-treated birds could be explained by an effect of lateralization, the FI between hemispheres in the vehicle-treated birds was also determined. The FI indices are not different between hemispheres in the vehicle animals (median left-NCM *F*I_vehicle_ = 1.122; median right-NCM *F*I_vehicle_ = 1.036; K-S *d*_stat_ = 0.162, *p* = 0.341) and the FI index was not different from 1.0 in either hemisphere (left-NCM *F*I_vehicle_, *p* = 0.154; right-NCM *F*I_vehicle_, *p* = 0.40; Bonferroni corrected).

### HDAC3-Inhibition Promotes Gene Expression Selectively in NCM

**Figure 4:**
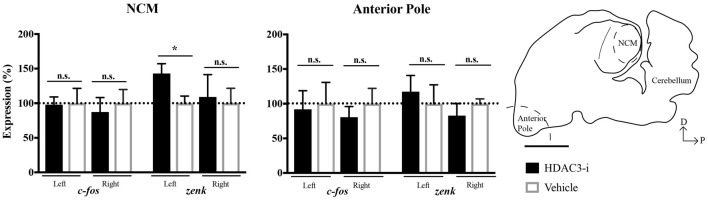
Effects of HDAC3-i on transcription demonstrated gene and region specificity. HDAC3-i induced significant increases in *zenk* (aka egr-1) expression selectively in left NCM with respect to vehicle controls. In contrast, this pattern of gene expression was not evident in *c-fos* expression (left panel; *zenk*: left NCM, *t*_(8)_ = 2.46, *p* = 0.039; right NCM, *t*_(8)_ = 0.232, *p* = 0.822; *c-fos*: left NCM, *t*_(8)_ = 0.083, *p* = 0.936; right NCM, *t*_(8)_ = 0.440, *p* = 0.672; one-sample *t*-test, Bonferroni corrected for the two hemisphere comparisons). This lateralized increased *zenk* expression was not observed in the Anterior Pole (AP, right panel; left AP, *t*_(4)_ = 0.479, *p* = 0.657; right AP, *t*_(4)_ = 0.919, *p* = 0.410; Bonferroni corrected for the two hemisphere comparisons). Schematized sagittal section of the zebra finch brain, sampled at 0.75 mm from the midline. The dashed curve line indicates the area dissected from the AP used in the gene expression study. NCM, caudal medial nidopallidum. Scale bar = 2500 μm. *Indicates significant difference between groups, alpha = 0.05.

To determine whether the effect of HDAC3-i to mediate neuronal memory in left NCM was due to changes in gene expression, the immediate early genes *c-fos* and *zenk* (i.e., *egr-1*) were selected for quantitative analysis suitable to detect differences between HDAC3-i and vehicle-treated birds trained with limited (20X) exposure. NCMs and the AP regions were collected from each bird 30-min after post-training injections. These genes were selected because *c-fos* is known to be regulated by HDAC3 (e.g., Malvaez et al., 2013) and this time point was chosen because of the known dynamics of *zenk* expression underlying the formation of exposure-dependent birdsong memory in zebra finches (Mello et al., 1995). HDAC3-i was found to significantly promote *zenk* expression selectively only in left NCM with respect to vehicle controls (one-sample *t*-test: left NCM, *t*_(8)_ 2.46, *p* = 0.039 ; right NCM, *t*_(8)_ = 0.232, *p* = 0.822; left AP, *t*_(4)_ = 0.479, *p* = 0.657; right AP, *t*_(4)_ = 0.919, *p* = 0.410; Bonferroni corrected for the two hemisphere comparisons). However, *c-fos* was not similarly affected (one-sample *t*-test: left NCM, *t*_(8)_ = 0.083, *p* = 0.936; right NCM, *t*_(8)_ = 0.440, *p* = 0.672; left AP, *t*_(4)_ = 0.196, *p* = 0.854; right AP, *t*_(4)_ = 0.723, *p* = 0.510; Bonferroni corrected for the two hemisphere comparisons; Figure 4). These findings indicate that HDAC3-i alters the regulation of *zenk* during the consolidation of birdsong memory. Moreover, the control of HDAC3 over gene expression appears to be lateralized with a left-side bias that is in agreement with the electrophysiological findings in NCM.

### HDAC3-i Enabled Neuronal Memory in NCM Partially Recapitulates the Memory Formed with Prolonged Exposure

**Figure 5:**
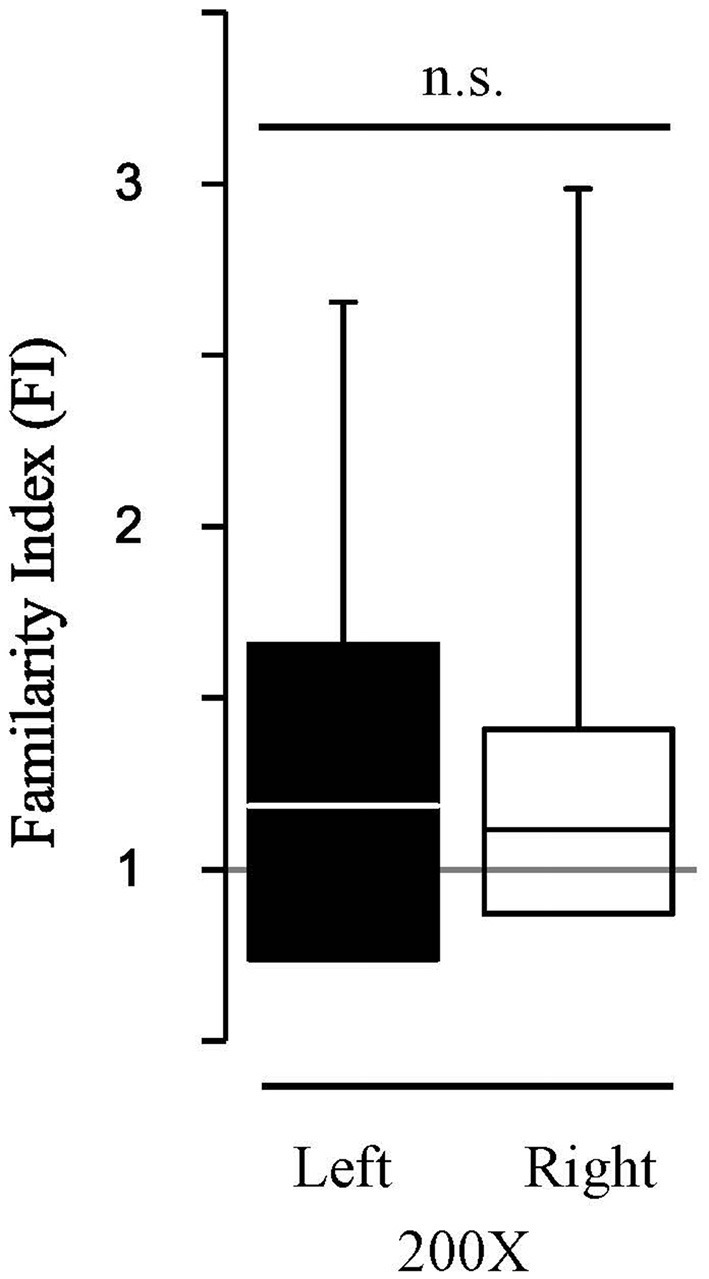
HDAC3-i coupled with the limited exposure paradigm (20X) reveals hemispheric differences in FI which is absent in the “typical” extended exposure condition (200X). The extended exposure paradigm (200X, no HDAC3-i condition) resulted in assessment of auditory memories that were more specific for previously exposed stimuli (Familiar) than for never before heard stimuli (Novel). However, in contrast, to the HDAC3-i treated birds, 200X birds do not show significant lateralized memory responses in FI (K-S *d*_stat_ = 0.159; *p* = 0.346). Figure conventions as in Figure 3.

To determine the extent to which the HDAC3-i enabled memory recapitulates “typical” memory without pharmacological treatment, the findings were compared to a new group of birds (*N* = 6) with extended song exposure (200X) alone. The 200X exposure did induce long-term memory; FI in 200X birds was significantly greater than 1.0 (*FI*_200X_, *Z*_signtest_ = 2.98, *p* = 0.0029). This effect of 200X exposure was the same as with limited exposure (20X) when treated with HDAC3-i (K-S d_stat_ = 0.692, *p* = 0.812). However, song-specific NCM neuronal memory is not lateralized with 200X exposure (Figure 5). Those birds show no hemispheric difference in FI (median left-NCM FI = 1.187; median right-NCM *FI*= 1.12; K-S *d*_stat_ = 0.159, *p* = 0.346). Thus, memory formed in NCM under natural conditions induced by 200X occurs without lateralization. Therefore, HDAC3-i enables memory formation from limited exposure (20X) that may induce a different form of neuronal plasticity from that which is formed in the 200X “natural” condition with extended exposure. These findings support that an HDAC3-selective pharmacological inhibitor permits the formation of song-specific neuronal memory in NCM after fewer exposures with effects that lateralize the representation of specific, salient sounds.

Overall, these results show that HDAC3 effects on memory extend to a systems-level change in experience-dependent plasticity that is selective both to a brain-region implicated in communication signal representation (NCM) and to one hemisphere (the left).

## Discussion

### Summary of Findings

The results indicate that an HDAC3-selective pharmacological inhibitor can enable the formation of song-specific neuronal memory in NCM. Birds treated with systemic administration of the HDAC3-i form neuronal memories for specific and unique conspecific songs after sub-threshold exposure of only 20 repetitions. This is a striking decrease in the threshold for memory formation in NCM. However, the results also indicated that memory facilitated by HDAC3-i only partially recapitulates naturally-induced neuronal memory with extended exposure. Remarkably, memories induced by HDAC3-i appear to be lateralized in NCM with a leftward bias. Lateralization is not evident with extended exposure alone (200X) or with limited exposure and vehicle treatment. Overall, these findings provide feasibility for the hypothesis that epigenetic mechanisms participate in a systems-level change in the neural encoding strategy for experiences with unique conspecific vocalizations.

### Epigenetic Mechanisms May Facilitate Remembering Highly Specific Sensory Experiences

Prior work has shown that epigenetic mechanisms can lower the threshold for the induction of long-term memory for behaviorally relevant cues, transforming a learning event from a short-term store into one that can last into the long-term ( >24 h) and even longer, beyond time points of those naturally formed. Stefanko et al. (2009) used a novel object recognition task in rodents to show failures of long-term memory formation as a result of reduced exposure time with novel objects. Remarkably, the failure can be rescued with an HDAC inhibitor, and furthermore, the long-term memory that forms lasts beyond the time-point at which a normal long-term memory would fail at 7 days. Until now, this finding has been a challenge to interpret as a simple change in the threshold for long-term memory formation beyond 24 h. The current findings suggest a novel interpretation of HDAC effects that extend memory (e.g., up to 7 days): animals with limited exposure but treated with an HDAC-inhibitor may have facilitated the amount of sensory detail that was encoded into memory. Thus, rather than interpreting a lower threshold for the induction of long-term memory as simply strengthening an existing collection of synapses that encode in parallel some subset of sensory features for a particular stimulus, HDACs could be controlling the way the whole configuration of specific sensory features—that represents the stimulus as a sensory object—is represented in the population as a memory over time. In this light, we interpret the lowered exposure threshold necessary for long-term memory induction in the present data as consistent with an epigenetic process (involving the HDAC3 system) that enables the larger configuration of sensory content to maintain memory over the long term. This conceptualization of the findings is in line with the hypothesis proposed by Phan and Bieszczad (2016), which states that epigenetic mechanisms gate the amount of sensory information encoded into memory after a relevant experience. In addition, recent evidence in rodents shows that auditory memories form with increased acoustic frequency-specificity when learned with HDAC-inhibitor treatment during the time of associative memory consolidation, which is reflected both in behavior and in the learning-induced plasticity of the auditory cortex (Bieszczad et al., 2015; Shang and Bieszczad, 2016). More generally, studies of epigenetic mechanisms including histone acetylation and DNA methylation also support this hypothesis (Day and Sweatt, 2011; Federman et al., 2014; Rudenko and Tsai, 2014; Spiegel et al., 2014). A role for epigenetic mechanisms in encoding specific sensory information has even been shown in associative odor discriminations in honeybees (Biergans et al., 2012, 2015, 2016). Together, these studies support an important evolutionarily conserved role for epigenetic regulation during salient learning experiences that extends across species and across sensory modalities to facilitate the amount of sensory detail that is encoded into memory.

### HDAC3-i Gates the Formation of Memory for Conspecific Song in NCM

SSA in songbirds presents a unique neuronal measurement for the maintenance of specific auditory memories over time. SSA is quantified as a reduction in neuronal responses to repeated presentations of an auditory stimulus however, the reduction (i.e., neural adaptation) is specific to each unique stimulus. The “stimulus-specific” aspect of SSA allows for the quantification of the degree in which one stimulus is remembered (e.g., familiar) over another (e.g., novel). The present study measured electrophysiological responses in NCM for SSA to song playbacks at a long-term time point (20 h) after the song exposures. NCM was targeted due to its known functions in the storage of song from the songbird’s tutor, often used as a model for juvenile song learning (Bolhuis et al., 2000; Terpstra et al., 2004; Phan et al., 2006; Gobes and Bolhuis, 2007; London and Clayton, 2008; Hahnloser and Kotowicz, 2010). Yet in the adult bird, this region also subserves song discrimination and stores memories for conspecific songs of other individuals (Mello et al., 1992; Thompson and Gentner, 2010; Tsoi et al., 2014; Soyman and Vicario, 2017) and is known to incorporate new neurons through experience-dependent neurogenesis throughout adulthood (Goldman and Nottebohm, 1983; for review: Pytte, 2016). That the neuronal memory for conspecific songs induced with HDAC3-i treatment occurred in NCM is in agreement with its known role in conspecific song memory and discrimination. Of course, while it is apparent that one aspect of a memory trace for experienced conspecific songs can be formed in NCM, this is unlikely to represent the entire memory for those songs, so other aspects of the memory may have traces elsewhere in the network of conspecific song-memory. Our findings are consistent with the hypothesis that HDAC3 normally puts the brakes on memory, here revealed by SSA, a neuronal mechanism that is naturally evident in the context of NCM function after optimal stimulus exposure conditions (Chew et al., 1996a,b; Tsoi et al., 2014). Thus, an inhibitor that releases the brakes will permit neuronal activities consistent with SSA under limited exposure conditions that normally do not result in long-term adaptation. The mechanisms that are under such control of HDAC3 directly in NCM neurons or indirectly elsewhere to induce SSA is an open question for future investigation. Indeed, since the present data were obtained with systemic injections, the site(s) of HDAC3-i action are unknown and the observations in NCM may be indirect. However, the hemispheric specificity of SSA in NCM suggests that HDAC3-dependent, NCM-specific processes are involved in neuronal memory.

### HDAC3 Elicits the Lateralization of Function for Encoding Birdsong Memories

Strikingly, the neuronal memory enabled in NCM by HDAC3-i was lateralized to the left hemisphere. Converging evidence from electrophysiological recordings (Phan and Vicario, 2010; Tsoi et al., 2014; Bell et al., 2015), quantifications of neurogenesis (Tsoi et al., 2014), immediate early gene induction (Avey et al., 2005; Moorman et al., 2012) and fMRI studies (Voss et al., 2007) support that song-selective processing in songbird NCM can be lateralized. Known factors that elicit song-evoked lateralization include age, song novelty, and task-specific demands. For example, juvenile auditory experiences during song learning modulates the degree of lateralization in the adult. Phan and Vicario (2010) manipulated the auditory environment of developing male songbirds by constraining their availability to hear the tutor song and motor ability to produce self-vocalizations. Whereas birds that can hear and imitate the tutor song show overall neuronal activity that is higher in right NCM than left NCM, lateral differences were absent in birds raised in acoustic isolation and with singing constraints. Furthermore, freely singing birds normally show less rapid adaptation in left NCM than in the right for the same song stimuli. Chirathivat et al. (2015) also reported that male birds raised in isolation without juvenile experiences with an adult conspecific song have overall fewer Zenk-immunopositive cells, which is in contrast to the typical increase in Zenk-immunopositive cells, more in left NCM than in right NCM after juvenile exposure to the learned tutor song (Moorman et al., 2012). These significant leftward asymmetries in juvenile songbirds suggest that leftward biases exist when the brain is in a “plastic” state of sensorimotor (imitative) learning during development. Learning-dependent asymmetries induced by song exposures disappear in mature songbirds that have already crystalized their adult songs (Moorman et al., 2012). Thus, because birds in the current study were adults, we did not expect to find that extended song exposure *per se* would produce lateralized effects, which was confirmed with 200X exposure in the current report and supported by work from Chew et al. (1996a,b). The emergence of lateralization in song-evoked activity enabled by HDAC3-i is of particular interest because it was unexpected.

In adulthood, only surprise or *novelty* in auditory environments has been shown to affect hemispheric lateralization of song-evoked responses. For example, Yang and Vicario (2015) reported that adult male zebra finches passively exposed to a hetero-specific sound environment (i.e., predominantly canary songs) evoke responses that are initially right lateralized. However, if exposure extends for 4 days, they eventually develop stronger evoked responses in the left hemisphere than the right—lateralization reverses. Thereafter, hemispheric lateralization is predicted to return to the native state (right-ward bias in evoked responses) when the hetero-specific environment is no longer novel with continued exposure. Nevertheless, experience-dependent reorganization of lateralization is not observed in birds exposed to conspecific sounds.

One important factor to consider with relevance to the current findings is the *strength of discriminative memory*. The development of memory for a particular conspecific song (vs. another conspecific’s song) *does* change hemispheric asymmetry. In long-term memory studies in adult zebra finches (>20 h after a song-learning experience), Tsoi et al. (2014) measured evoked response magnitudes to conspecific songs to calculate a relative response strength measure which quantified the magnitude of evoked response for the previously heard songs (F), relative to the never-before-heard songs (N). This metric revealed that the relative magnitude of responses to only the *remembered* songs was higher in the left than the right hemisphere. This result is entirely consistent with the current findings for HDAC3-i treated birds, which also show lateralization in the neuronal memory measured by relative differences in adaptation rate for the exposed song stimuli. It cannot, however, explain the lack of lateralized neuronal memory in the 200X-treated birds. Therefore, we interpret these data to mean that the memory for song induced with extended experience (200X) is qualitatively different from the HDAC3-i-enabled memory induced with only limited experience (20X). The qualitative difference may be in the formation of *discriminative memory*, which involves the amount of discriminative acoustic-feature information that a bird has available to recall from memory if a newly heard song is “familiar” (remembered) or “novel” (forgotten). The induction of cerebral asymmetry may be related to the apparent discrimination ability for subsequently heard sounds. In support of this interpretation, the direction of the leftward bias in birds treated with HDAC3-i is in agreement with prior published correlative findings between experience-dependent neural plasticity and discriminative learning ability (Cynx et al., 1992). For example, Bell et al. (2015), reported that the rate that zebra finches learn go/no-go discriminations of song sounds is related to the direction of lateralization of auditory responses in NCM—faster learners had greater evoked responses in the left hemisphere, slow learners in the right. Together, these findings suggest that future studies should investigate the differences in song discriminability for birds trained with extended exposure (i.e., 200X without HDAC3-inhibitors) vs. those trained and treated with HDAC3-inhibitors. If HDAC3-i enables more of the identifying acoustic features of a single conspecific song to enter into long-term memory than natural long-term memories induced by extended exposures, then HDAC3-i treatment will yield birds that are better able to discriminate between two similar conspecific songs for recall after training. Nonetheless, the current findings establish feasibility for a molecularly-driven lateralization effect on a systems-level reorganization of neural responses to salient sounds.

### Molecular Mechanisms of Unique Songbird Memories in Zebra Finches

The present findings show that epigenetic mechanisms may control the neural plasticity underlying the formation of specific memories for conspecific communication sounds. Furthermore, we demonstrate that pharmacological inhibition of HDAC3 is linked to experience-dependent genes that are known to be targeted for expression in song-specific memory formation. Here, we found that in the expression of the *zenk* gene (also known as *zif268*, *egr-1*, *ngfi-a* and *krox24*), which is an immediate early gene often used to indicate select populations of neuronal activation for stimulus- and event-specific experiences (Mello et al., 1992, 1995; Mello and Clayton, 1994; Bolhuis et al., 2000; Terpstra et al., 2004). Expression of *zenk* is increased in HDAC3-i treated birds, relative to vehicle-treated controls with limited exposure which fail to have induced neuronal memory. In contrast, the reason for the lack of detectable effects on *c-fos* may be due to the failure to capture its expression at a critical peak time-point during the consolidation of birdsong memory. For example, these studies investigated a 30-min time point (which is a known peak in expression for *zenk*, Mello and Clayton, 1994; Chew et al., 1996a,b; Jarvis and Nottebohm, 1997). However, *c-fos* may be differentially regulated by HDAC3-i at a later time point, e.g., at 1 h, as was shown for rodents treated with RGFP966 in Malvaez et al. (2013). Future studies may begin to link epigenetic determinants with their many downstream effects to orchestrate the temporal and spatial dynamics of gene expression events *in toto*. Such epigenetic links to gene expression in the avian brain were first supported by Toporova et al. (2016), who reported that class I HDACs regulate local *c-fos* and *zenk* expression in select brain regions to support associative learning in object avoidance tasks.

## Conclusion

Broadly, these data suggest a molecular basis for experience-dependent neural plasticity, memory storage and the lateralization of function for sensory representation that may be regulated by epigenetic mechanisms. Furthermore, these findings support the interpretation that each hemisphere’s brain region-specific representation of conspecific songs may be functionally relevant for how the distinctive acoustic features of unique vocalizations are remembered.

The findings are also relevant for understanding how naturally salient sensory events, like frequent exposure to conspecific communication sounds, may have unique access to memory encoding and storage. There may be epigenetic regulation of acoustic social communication events to learn and remember highly specific and behaviorally significant acoustic signals. More generally, the findings suggest epigenetic processes may contribute not only to memory formation that *remains robust* with time (e.g., McQuown and Wood, 2011; Federman et al., 2014), but also that *maintains distinctive sensory features*, which makes sensory information available later for discriminative memory recall (Phan and Bieszczad, 2016). The present data can be interpreted to relate to other interpretations of HDAC function found in the literature, such as directing a change in the threshold to form a long-lasting memory; inhibiting HDAC3 may enable long-lasting memory by facilitating the precision and quantity of sensory details that are encoded into memory. Finally, these data provide initial evidence for HDAC3 as a candidate molecular target for the development of therapeutics that aid auditory memory formation. Its application could be for naturally salient communication sounds in individuals with speech processing or acoustic learning disorders that would benefit from gaining the ability to remember the highly specific acoustic details of communication sounds.

## Author Contributions

MLP and MMG contributed equally as co-first authors. MLP, KMB and DSV: conceived and designed the experiments. MLP, MMG, SM and JJ-C: performed the experiments. MLP, MMG and KMB: analyzed the data. MLP, MMG and KMB: contributed to the writing of the manuscript. DSV, SM and JJ-C: contributed to the editing of the manuscript.

## Conflict of Interest Statement

The authors declare that the research was conducted in the absence of any commercial or financial relationships that could be construed as a potential conflict of interest.

## Footnotes

^1^http://www.zebrafinchatlas.org/
